# Insulin-Like Growth Factor-1 (IGF-1) Deficiency and Metabolic-Dysfunction-Associated Steatotic Liver Disease in a Young Patient

**DOI:** 10.7759/cureus.77146

**Published:** 2025-01-08

**Authors:** Christina Flourou, Chara Azina, George Georgiou, Violetta Anastasiadou

**Affiliations:** 1 Department of Internal Medicine, Nicosia General Hospital, Nicosia, CYP; 2 Department of Histopathology, Nicosia General Hospital, Nicosia, CYP; 3 Genetics Clinic, Karaiskakio Foundation, Nicosia, CYP

**Keywords:** cirrhosis, growth hormone (gh), insulin-like growth factor-1 (igf-1), liver fibrosis, steatotic liver disease (sld)

## Abstract

Metabolic-dysfunction-associated steatotic liver disease (MASLD) is the most common chronic liver disease in the Western world. MASLD-associated cirrhosis prevalence is on the rise along with the obesity and metabolic syndrome epidemic. Genetic factors are included in the multi-hit model of MASLD pathogenesis and insulin-like growth factor-1 (IGF-1) has an important role.

We report the case of a man who was referred to a hepatology clinic due to elevated liver enzymes as probable drug-induced liver injury (DILI). A 35-year-old man was diagnosed with compensated cirrhosis with an estimated Child-Pugh score of 5 points (Class A) and underwent further investigation of the causative factor. MASLD-cirrhosis was the preliminary diagnosis, but high serum and urine copper levels needed further investigation. Whole-genome sequencing revealed heterozygosity for a rare variant of the IGF-1 receptor, a metabolic factor whose role is crucial in the GH/IGF-1 axis to fatty liver and cirrhosis.

MASLD diagnosis is really challenging, especially at the progressive stages of fibrosis. Clinical features, somatometric parameters, laboratory tests and liver biopsy guide us to establish the diagnosis. Despite all these findings, the heterogeneity of disease’s pathogenesis through metabolic pathways underlines the need for deeper investigation, especially genetic factors such as IGF-1 and their penetration in disease progression and liver fibrosis.

## Introduction

Steatotic liver disease (SLD) is the most common chronic liver disease and includes all the etiologies related to fatty liver infiltration such as metabolic syndrome, alcohol consumption, drugs, monogenic diseases, and genetic disorders [1]. Metabolic-dysfunction-associated steatotic liver disease (MASLD) is the most common category of SLD which is associated with metabolic syndrome epidemic and cirrhosis progression. The heterogeneity of disease’s pathogenesis makes the differential diagnosis difficult, especially in young patients.

This case presents a patient with liver injury referred as a drug-induced liver injury (DILI), but compensated cirrhosis diagnosis deteriorated the investigation.

## Case presentation

A 35-year-old man was referred to the hepatology unit due to elevated liver enzymes. He had a past medical history of hypertension. He was a nonsmoker and social drinker, 1 beer weekly, and he refused over-the-counter supplements. He received treatment with terbinafine for the last four weeks, and he was referred for probable DILI.

On clinical examination, he had no neurological defects or jaundice, but he had spider nevi, palmar erythema, and hepatomegaly with no splenomegaly or ascites.

Phenotypically, the patient had microcephaly, short stature with a height of 155 cm, and obesity stage 1 with an estimated BMI of 35 kg/m^2^. Anthropometry parameters of neck circumference (40 cm), waist circumference (125 cm), and waist-to-hip ratio (1.04) measurement confirmed obesity. 

The first laboratory workup showed elevated liver enzymes where alanine and aspartate transferases were two times the upper normal limit (UNL) and γ-GT was three times the UNL (Table 1).

**Table 1:** Laboratory parameters AFP: Alpha fetoprotein; ALT: alanine transaminase; SLA-LP: soluble liver antigen/liver-pancreas; SMA: smooth muscle antibody

Variable	Reference range	At presentation
Hemoglobin (g/dl)	13-15	14.7
Hematocrit (%)	40-54	42.8
Mean corpuscular volume (fL)	77-93	100
White blood cells (x10^9^/l)	3.9-8.9	9.38
Neutrophils (x10^9^/l)	1.8-7.2	5.18
Lymphocytes (x10^9^/l)	0.85-3.0	3.26
Monocytes (x10^9^/l)	0.2-0.7	0.73
Eosinophils (x10^9^/l)	0.03-0.4	0.18
Basophils (x10^9^/l)	0.01-0.05	0.03
Platelets (x10^9^/l)	150-450	219
INR	0.9-1	0.9
Total bilirubin (μmol/l)	0.3-1.2	0.7
ALP (U/l)	30-120	59
γ-GT (U/l)	9-55	150
ALT (U/l)	3-40	85
AST (U/l)	3-40	90
LDH (U/l)	208-480	350
Ferritin (ng/ml)	20-300	350
Total protein (g/dl)	6.6-8.4	8.4
Albumin (g/dl)	3.5-5.2	3.5
Total cholesterol (mg/dl)	<200	180
LDL (mg/dl)	<130	92
HDL (mg/dl)	>40	52
Triglycerides (mmol/L)	50-150	180
HbA1c (%)	4-6	5.8
IgG (mg/dl)	700-1600	1400
IgM (mg/dl)	40-230	95
IgA (mg/dl)	70-400	460
TSH (mIU/L)	0.5-5	2.7
Ceruloplasmin (mg/dl)	20-60	32
Serum copper (mcg/dl)	63–158	190
24-h urine copper (μg/L)	2-65.0	128.0
a1-Antitrypsin (mg/dl)	90-200	180
ANA		Negative
ANA specific (gp210, sp100)		Negative
SMA		Negative
pANCA		Negative
SLA-LP		Negative
anti-LKM1		Negative
anti-LC1		Negative
AFP	<7.0	3.0

Serological tests for hepatotropic viruses (Ebstein-Barr, Cytomegalovirus, Varicella-zoster, Herpes simplex 1 and 2, Hepatitis A, B, C, E) were negative, so acute or chronic viral hepatitis was excluded. Autoantibody testing for autoimmune hepatitis through indirect immunofluorescence on triple tissue (rodent liver, kidney, and stomach) was negative for autoimmune hepatitis. Elevated serum immunoglobulin A (IgA) was observed with normal ranges of IgG and IgM. Serum protein electrophoresis revealed no paraproteinemia.

Alpha-1 antitrypsin enzyme and ceruloplasmin levels were in the normal range although serum and 24h urine copper levels were both elevated. His investigations are shown in Table 1. High serum and 24h-urine copper levels required further investigation. Slit lamp examination did not show the presence of Kayser-Fleischer rings. Whole-genome sequencing (WGS) was performed to examine the presence of ATP7B gene mutation which was not found. Thus, Wilson’s disease was excluded.

**Figure 1:**
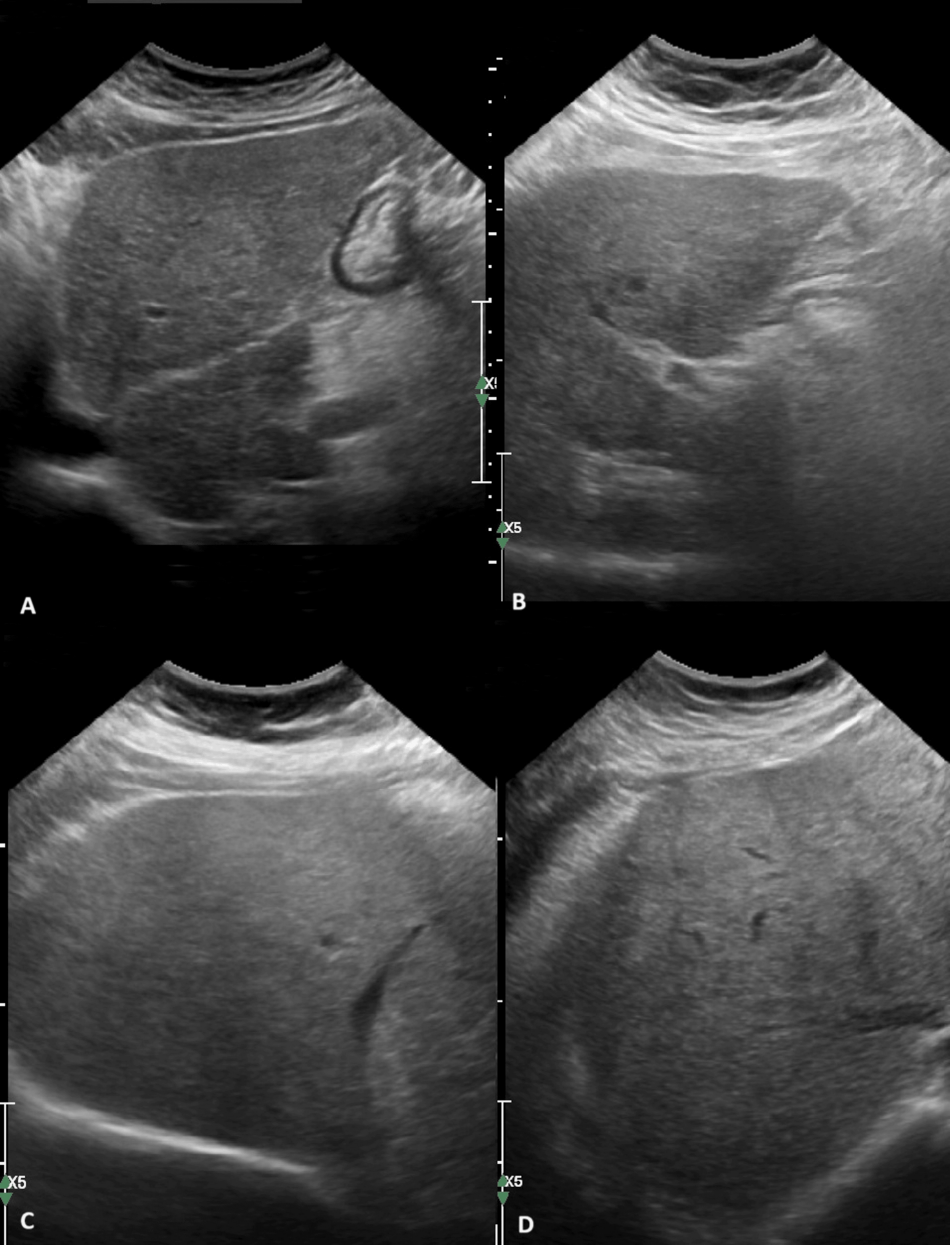
Ultrasound of the abdomen revealed hypertrophy of the left lobe (A, B) and hepatomegaly (C, D) with surface nodularity. Ascites was not observed.

Ultrasound of the abdomen revealed hepatomegaly with surface nodularity and hypertrophy of the caudate lobe. No splenomegaly or ascites was noticed. Hepatic vessels, portal vein, and splenic vessels were patent (Figure 1). The portal vein diameter was 10mm and no portosystemic shunts were observed. At the same time, liver elastography measured liver stiffness at 15kPa indicating liver cirrhosis stage F4. Non-invasive biomarkers such as FIB-4 at 1.56 points confirmed the advanced fibrosis stage which required further investigation.

**Figure 2:**
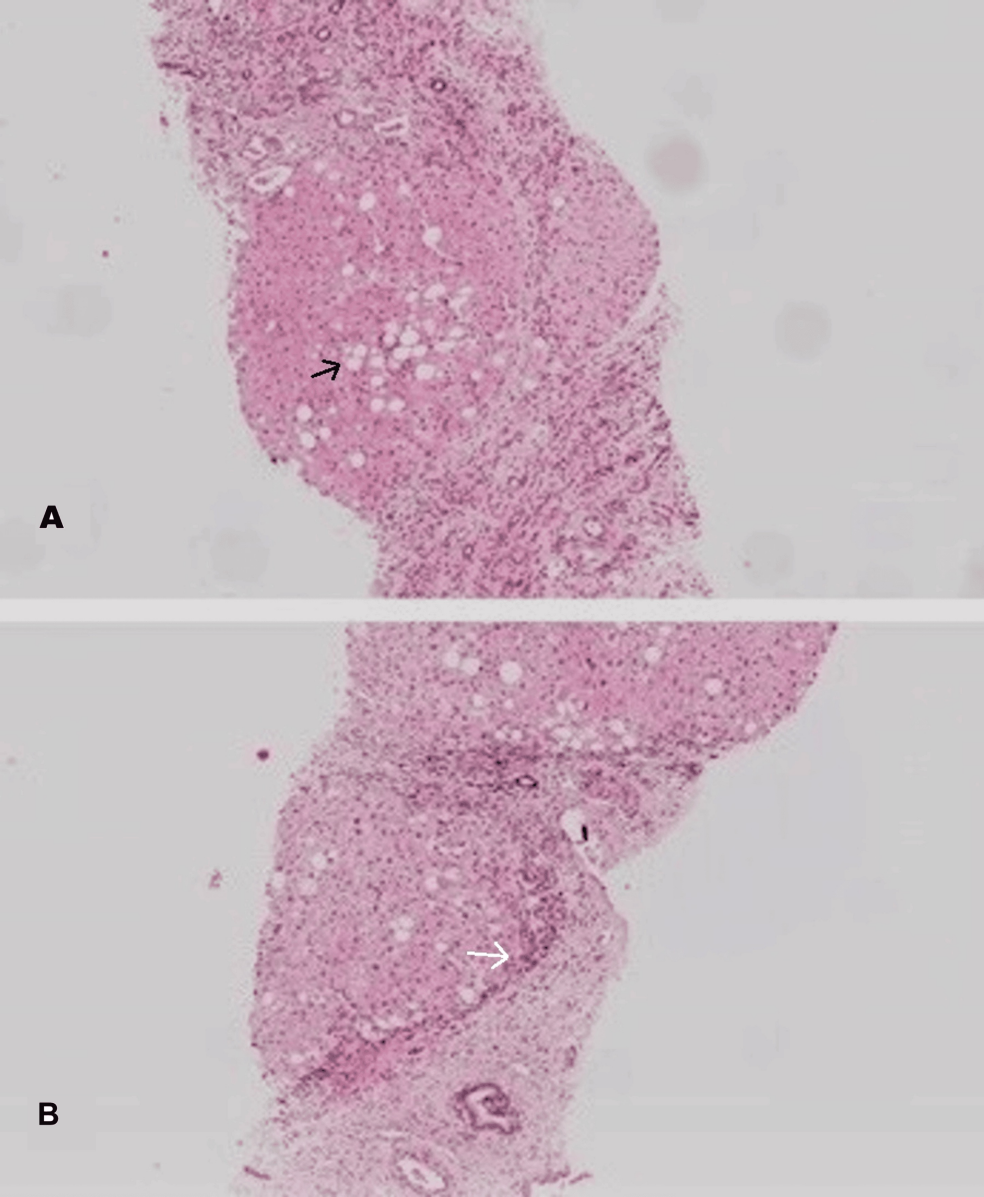
Hematoxylin and eosin staging showing a steatotic pattern with diffuse involvement of the lobular parenchyma by fat droplets (black arrow). Fibrotic bands are observed and distort the liver parenchyma (white arrow).

Liver biopsy was performed to find the causative factor of cirrhosis. Histological assessment revealed regenerative nodules of liver parenchyma and dilated portal tracts with fibrous connective tissue (Figure 2). Notably, more than 30% of hepatocytes presented macrovesicular steatosis, ballooning degeneration, and Mallory-Denk bodies. Orcein and Congo red stains were negative for copper and amyloid deposition. 

Impressively, the WGS identified heterozygous Arg74Cys (c.220C>T) mutation in the IGF1R gene which is a rare variant. The mutation in IGF-1R was added as a co-factor in liver fibrosis progression manifesting cirrhosis in our young patient in the context of metabolic dysfunction liver disease. A family screening was also performed.

The patient fulfilled the criteria for MASLD diagnosis. To clarify these criteria, hepatic steatosis was confirmed through imaging and histology, significant alcohol consumption (30-60 g/day for males) was excluded according to his medical history and he was obese with a BMI over 25 kg/m^2^. Additionally, the presence of cardiometabolic risk factors such as hypertension and high levels of plasma navigated the diagnosis. Lifestyle modification strategies were suggested to the patient such as weight reduction by caloric restriction, physical activity, and exercise. Pharmacological therapy with high-intensity statins combined with fibrates prescribed, rosuvastatin 40mg/fenofibrate 145mg and vitamin E 800 IU as a non-diabetic patient was suggested. Glucagon-like peptide 1 receptor agonist (GLP1-RA) semaglutide is suggested following the latest recommendations [2].

## Discussion

The patient in our study was referred to the hepatology clinic for evaluation due to transaminitis as a possible DILI presentation. Idiosyncratic DILI has multiple clinical and laboratory expressions although it is a diagnosis of exclusion and requires careful assessment. Clinical, imaging, and histopathological evaluation confirmed compensated cirrhosis and redirected the investigation. The patient had SLD due to MASLD because he was obese with at least three metabolic abnormalities: high waist circumference, arterial hypertension, and high plasma triglycerides [3]. In SLD investigation, all the causes of secondary fatty liver infiltration should be excluded such as alcohol consumption (20-50 g/day for women and 30-60 g/day for men, respectively), viral hepatitis, hypobetalipoproteinemia, celiac disease, and Wilson's disease [4].

High serum and urine copper needed further investigation, to exclude Wilson's disease as a cause of secondary fatty liver disease in young patients. The patient did not fulfill the diagnostic criteria of Leipzig for Wilson’s disease, but copper is an established contributor factor in liver fat accumulation and inflammation [5]. Obviously, the homeostasis of copper plays an important role in lipid metabolism. Copper attenuates liver steatosis in two different ways: first, by inhibition of PDE3B, an enzyme of lipolysis, and second, by lipogenesis enhancement through the activation of the Nrf2-PPARγ pathway [6,7].

The liver has a central role in glucose and lipid homeostasis and through its endocrine function participates in endocrine factor secretion such as insulin-like growth factor-1 (IGF-1), as well as its binding proteins [8]. The GH/IGF-1 metabolic pathway has correlated with hepatocyte triglyceride secretion, liver steatosis, and fibrosis progression [9]. In experimental studies in fatty liver-induced models, the animals had decreased levels of IGF-1, while opposite animals treated with GH and IGF-1 had reduction of liver triglycerides and transaminases [10]. At the same time, in animal models with steatohepatitis induced by the methionine-choline-deficient diet, histological improvement of hepatocyte morphology and inflammation was observed after IGF-1 administration [11]. Notably, IGF-1 has an anti-inflammatory effect on hepatocytes by reducing oxidative stress, mitochondrial dysfunction, and apoptosis [11].

Despite its role in liver steatosis, IGF-1 has a key role in fibrinogenesis, cirrhosis, and hepatocellular carcinoma development through stimulation of liver degeneration and tissue repair [12]. Lower levels of circulating IGF-1 showed lower survival rates in patients with cirrhosis than those with high plasma IGF-1 levels [13]. Additionally, IGF-1 levels are a more sensitive prognostic factor for the disease progression to decompensation in comparison with other conventional parameters such as albumin [13]. Interestingly, the conventional scores which are used widely for prognosis as MELD and Child-Pugh scores predict short-term mortality in end-stage liver disease and differences in prognosis between patients in classes A, B, and C, respectively, but these scores are limited in patients with compensated cirrhosis. However, through the studies, IGF-1 is described as a predictor of long-term prognosis in the compensated stages [13].

To date, the WGS revealed a very rare mutation of the IGFR1 gene. This variant has not been described yet.

## Conclusions

In summary, the GH/IGF-1 axis has a crucial role in metabolic and endocrinal pathways of liver steatosis while IGF-1 deficiency is established as a co-factor to this syndrome. The heterogeneity of disease’s pathogenesis underlines the need for deeper investigation, especially genetic factors and their penetration in disease progression. Despite the presence of obvious metabolic risk factors to establish the diagnosis of MASLD-cirrhosis, other causes should be detected especially in young patients with rapid fibrosis progression. Overall, this case underlines the need for a multidisciplinary approach in investigation, diagnosis, and treatment in advanced liver fibrosis stages especially, in a young population.
